# The Effects of Winter Parks in Cold Regions on Cognition Recovery and Emotion Improvement of Older Adults: An Empirical Study of Changchun Parks

**DOI:** 10.3390/ijerph20032135

**Published:** 2023-01-24

**Authors:** Tianjiao Yan, Hong Leng, Qing Yuan

**Affiliations:** 1 School of Architecture, Harbin Institute of Technology, Harbin 150001, China; 2 Key Laboratory of Cold Region Urban and Rural Human Settlement Environment Science and Technology, Ministry of Industry and Information Technology, Harbin 150001, China

**Keywords:** winter parks, cold regions, older adults, cognition recovery, emotion improvement

## Abstract

Urban parks are one of the primary settings for older adults to exercise, and their health benefits have been confirmed by a large number of studies. However, with the increased social attention to mental health, there is not enough research on the short-term mental health recovery of older adults in parks. Meanwhile, the health recovery effects of winter parks in special climate areas have not been well explored. This study aimed to explore the effects of winter parks in cold regions on the short-term mental health recovery of older adults and the potential predictors of these effects, including individual status, park characteristics, and behavioral characteristics. This study divided short-term mental health recovery into cognitive recovery and emotional improvement, and selected the digit span test and 10 kinds of emotional expression as the experimental methods, recruited 92 older adults from 6 parks in Changchun, and compared the pre-test and post-test results for evaluation. The results showed that winter parks in cold cities still had short-term cognitive recovery and emotional improvement effects on older adults. The main park characteristic factors affecting the overall cognitive recovery were the evergreen vegetation area and the existence of structures, and that which affected the overall emotional improvement was the main pathway length. Furthermore, individual conditions, including gender, age, physical health, living and customary conditions, and park characteristics, including park type, park area, main pathway length, square area, equipment area, evergreen vegetation area, the presence of water, and structures, all related to short-term mental health recovery effects. Among behavioral characteristics, stay time in parks and MVPA (Moderate and Vigorous Physical Activity) times were also related to certain effects, but behavior type was not.

## 1. Introduction

China has entered an aging society and will become one of the fastest-aging countries [1,2]. From 2010 to 2040, it is estimated that the proportion of China’s population over 60 years old will increase from 12.4% to 28%. It is estimated that by 2050, China will become a world giant with 320 million older adults [3,4]. At present, while facing the challenge of aging, the health problems of older adults are also ignored. The major health problems faced by them in China are diseases caused by cardiovascular and mental health problems (such as depression and Alzheimer’s disease) [5]. Among them, mental health problems have attracted more and more attention from all walks of life. The current situation of mental health among older adults in China, jointly published by the Institute of Psychology of the Chinese Academy of Sciences and the Social Sciences Literature Press, expresses that nearly one third of older adults in China have depression, and the overall prevalence of MCI (Mild Cognitive Impairment) among them over 65 is 20.8% [6]. It also points out that outdoor exercise is good medicine for the recovery of the mental health of older adults.

The role of urban parks in promoting residents’ physical and mental health has long been the consensus of researchers. Among them, the potential for mental health recovery in parks includes both long-term and short-term effects. The former is that residents who live closer to parks and interact with parks more have better mental health performance [7]. The latter is that urban parks can help tourists restore perception, relieve pressure, and improve mood through natural elements or places and opportunities for sports activities and social interaction [8,9,10,11]. These recovery potentials have been explained by theories of restorative environments, such as the Stress Reduction Theory (SRT) [12] or the Attention Restoration Theory (ART) [13].

As the travel circle and mode of older adults are relatively fixed, parks have become the most frequently selected space carrier close to nature. Older adults visit parks more frequently than young people [14]. Some studies showed that the mental health of the elderly living near the park was better due to the promotion of sports and social activities and the provision of a good ecological environment and communication space [15,16,17]. However, few studies have explored the relationship between park characteristics and the short-term psychological recovery effect on older adults.

For older adults, the short-term psychological recovery effects of green space include cognitive recovery and emotional improvement [18]. These are also the keys to solving the prominent psychological problems of older adults mentioned above. The existing research methods mainly focus on observation, while the experimental methods have gradually increased in recent years [19], including laboratory experiments and field experiments. The former carries out a perception recovery test and physiological index measurement [20,21,22] in the form of VR, while the latter measures physiological, attentional, and emotional indexes before and after the experiment in the form of field roaming [18,23,24]. The physiological indicators are mainly blood pressure, heart rate, EEG (Electroencephalogram), et al. [25,26]. Cognitive recovery experiments are usually carried out in the laboratory, using methods such as the digital span test (DST) [27], short cognitive reaction time (SRT) test [18], and NCPC (Necker Cube Pattern Control) task [24]. Russell [28] proposed a circumplex model for emotion classification, and he believed that emotions can be divided into two dimensions: pleasure and arousal. The Edinburgh Mental Well-Being Scale [29], SF-8 [30], POMS (Profile of Mood States) and PANAS (Positive and Negative Affect Schedule) [31,32] are often used to measure emotion, including confusion, vigor, fatigue, anger, tension, and depression. A few studies also selected or increased the expression of targeted emotional dimensions [26]. For example, in Church’s et al. [33] study, residents’ perceptions of relaxation, enjoyment, and revitalization after park use were measured. Wolf and Wohlfart [34] measured the levels of health and well-being improvements after park use. As for technical methods, data acquisition methods have become diversified, such as SOPARC (System for Observing Play and Recreation in Communities) [35] and GPS trajectory [31,36]. Although there are more and more relevant research methods, there are few experiments [20,23,25,26,36] on elderly adults in general, and their particularity is not considered enough.

Relevant studies analyzed the recovery effect of parks on individual physiology, cognition, and emotion from the aspects of individual status, space type and characteristics, and use behavior [31,36]. Firstly, scholars confirmed that differences in individual attribute characteristics, such as gender, age, residence status, physical health, economic income, and education level, can affect their activity behavior and health status [30,31,37,38,39,40]. Secondly, some academics have also focused on the impact of spatial types and characteristics on the mental recovery effect [21,22,23,31]. Some studies have compared the natural environment with the built environment and found that older adults prefer the natural environment [20]. Moreover, the more natural the features, the better their recoverability [23,41]. In terms of spatial characteristics, a few scholars believed that environmental recoverability was usually affected by park scale and park characteristics [7,42]. They paid more attention to indicators based on physical activity levels, such as trail length, the square of a certain scale, equipment area, and the presence of water [7,43,44,45]. Thirdly, in the aspect of user behavior, activity duration and intensity were given more attention [31,46]. The behaviors in parks can be divided into passive, active and mixed according to spontaneity [47]; low, medium, and high-intensity behaviors by exercise intensity [31]. However, it is worth noting that the exploration of space and behavior characteristics in different regions and seasons is not deep enough.

To summarize, there are two obvious problems with the current research on the restorative effects of older adults and parks. On the one hand, empirical research in the field of mental health is not deep enough [48], especially with respect to methods with strong usability for older adults. On the other hand, the existing research lacks the excavation of special climate areas. For example, in winter cities, the extreme climate will indeed limit the travel range and modes of older adults to a certain extent, but their biophilia will not be reduced, and it is unknown whether parks have restorative effects on the mental health of older adults in winter.

Therefore, this study started from the point where parks meet the mental health recovery of older adults, that is, the attention and emotion experiment, and then focused on the cold climate background and tried to answer the following two questions: 1. In winter, do parks in cold regions still have short-term mental restorative effects on older adults? 2. What potential factors will lead to these effects?

## 2. Materials and Methods

### 2.1. Study Sites

**Figure 1:**
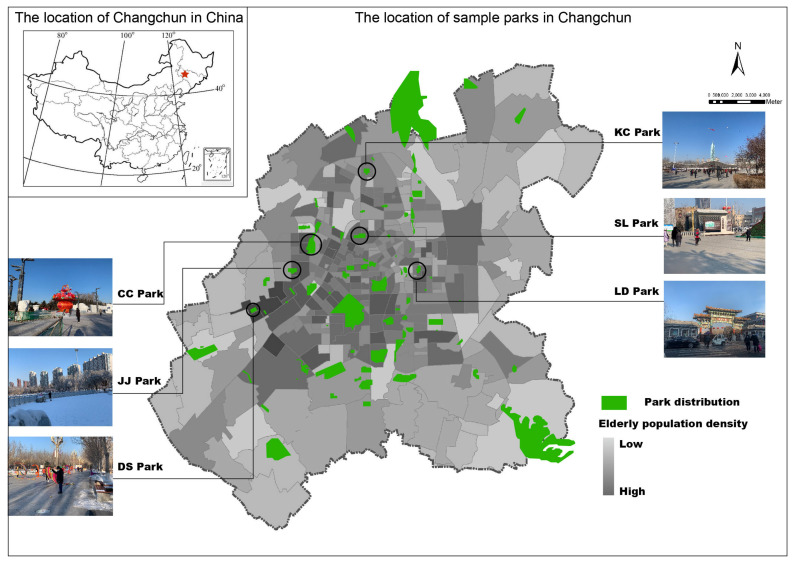
Site selection of sample parks. (Source: Modified from the data provided by Changchun Urban and Rural Planning and Design Institute.)

Changchun is a typical city in the severe cold regions of China, with an area of about 24,744 km^2^ and a population of about 9.07 million. It is worth noting that the aging problem in Changchun is serious. According to the seventh census data of Changchun Municipal Bureau of Statistics, by 2020, the population over 60 years old in Changchun will have reached 1.89 million, accounting for 20.85% of the total population. Parks with activities for older adults are mainly concentrated in the central area of Changchun. The areas with a high density of elderly population and all medium-sized and non-theme parks were investigated. Among them, six parks meeting the requirements were selected as research sites. Changchun Park, Shengli Park, Laodong Park, Daishan Park, Jinjiang Park, and Kuancheng Central Park were selected and abbreviated as CC, SL, LD, DS, JJ, and KC parks, respectively. These abbreviations were used below (Table 1 and Figure 1).

### 2.2. Study Procedure

Researchers recruited participants at the main entrances of these parks. The selection criteria were: older adults over 60 years old; no communication barrier or intellectual deficiency; and older adults who are prepared to use parks rather than just pass through. The research process was as follows: first, on the premise of consulting the consent of senior participants, we explained the research process, issued GPS locators, and tested their cognitive and emotional performance, then let them carry out nonintervention activities in these parks. After the activities, they took back the instruments and asked them to tell their exercise content; at the same time, they acquired the cognitive and emotional results again and finally recorded the basic personal information and gave small gifts. Each subject carried a GPS handheld locator to complete the whole process of the park visit, and a complete track record was formed at the end of the experiment. Based on the pace, accurate location, and other information obtained, this study can further determine the behavioral characteristics of each subject in combination with interviews.

Data collection was carried out on sunny days in January and March 2022 (average temperature was about −11 °C), which have common climate characteristics of cold cities in winter. The survey period was from 8:00 a.m. to 11:00 a.m. because older adults most often used parks during this period.

### 2.3. Measures

#### 2.3.1. Cognition and Emotion Measurements

**Figure 2:**
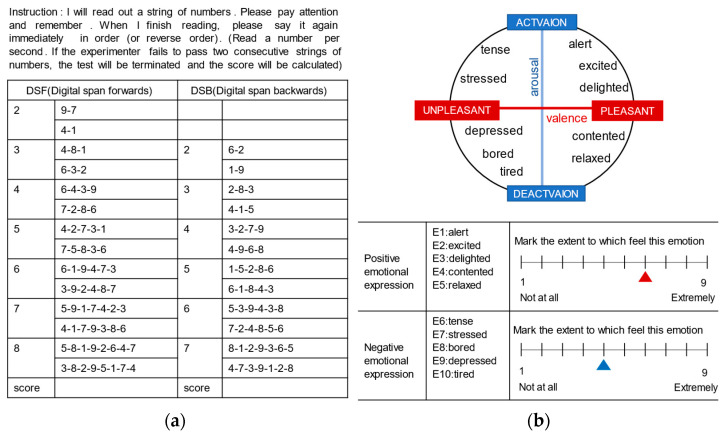
(**a**) Cognition test method; (**b**) Emotion measurement method.

The digit span test was chosen to measure attention, including the forward digit span (DSF) and backward digit span (DSB) tests. Testers recorded the test numbers in advance, and the subjects repeated the numbers according to the audio. The specific guide words and numbers are shown in Figure 2a. If they failed twice in succession, testers stopped the test and scored. Finally, this study evaluated the cognitive recovery level of the parks by comparing the scores of the test before and after entering the parks.

Based on POMS and PANAS, considering Russell’s circumplex model and combined with the degree of pleasure and arousal of emotions, 10 common emotional expressions were selected, including positive emotions, such as “alert”, “excited”, “delighted”, “contented”, and “relaxed”, and negative emotions, such as “tense”, “stressed”, “bored”, “depressed”, and “tired”. Furthermore, the experimental scheme was further improved by combining a visual simulation evaluation method [49,50], and gave a score of 1–9 for the degree of feeling various emotions (Figure 2b). 1 means not at all, and 9 means extremely. Similarly, this study used the emotional differences of older adults before and after entering the parks to evaluate the level of emotional improvement in these parks. Moreover, a validity test was conducted. The KMO is 0.714, indicating that the validity is high and can be further analyzed.

#### 2.3.2. Determination of Park Characteristics and Behaviors

**Figure 3:**
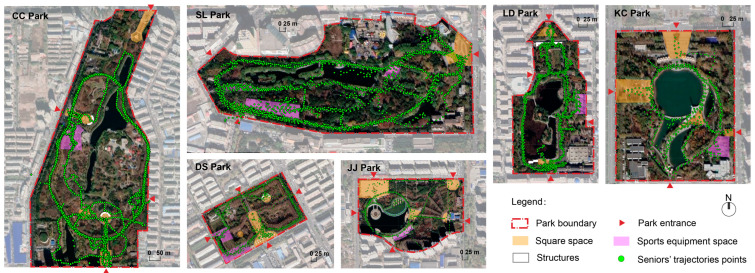
Maps of sample parks and older adults’ sample trajectories.

In the study of Zhai, et al. [31] in non-winter, older adults respectively spent 35.9% and 11.2% of their park visit time on pathways wider than 3.5 m and pathways narrower than 3.5 m, and 25% of their stay time on open squares more than 1000 m^2^. Therefore, this study used these two standards for statistics. And natural space area, the presence of water and equipment space were also very important in that study. For winter, evergreen vegetation area (evergreen trees were calculated with the crown width of 1 m) was used instead of natural space area as the potential impact factor. In addition, the results in behavior observation showed that such behaviors as playing cards and chess are mainly relied on structures. Therefore, the presence of water and structures, the total length of pathways (width >3.5 m), the total square area (>1000 m^2^), the fitness equipment space area, and the evergreen vegetation area were selected as park characteristic indicators (Table 2 and Figure 3). These indicators were obtained through data query of garden and field survey.

Behavior data was obtained through trajectories and supplemented by interviews, including the division of behavior types and the measurement of stay time and total MVPA time (Table 2). According to the purpose and content, the behaviors of older adults can be divided into fitness behaviors, entertainment behaviors, and leisure behaviors. About exercise intensity, this study combined the concept of MET, i.e., the ratio of work metabolic rate to rest metabolic rate. For example, walking is estimated to be 3 METs and running is estimated to be 6 METs. The behavior associated with MET > 3 is usually defined as moderate and vigorous physical activity (MVPA) [31,51,52].

#### 2.3.3. Statistical Analysis

IBM SPSS statistical software (Version 20.0, IBM, Armonk, NY, USA) was used for statistical analysis. Descriptive statistics were used to determine the characteristics of the collected sample data and the design characteristics of these parks. Furthermore, categorical variables and continuous variables were distinguished, and the differences of recovery effects of various personal characteristics, park characteristics, and behavioral characteristics were tested by independent sample *t*-test, Pearson analysis, and one-way ANOVA. Furthermore, this study integrated the scores of DSF and DSB test to evaluate the overall situation of cognitive recovery and used “(Δ positive emotion–Δ negative emotion)/10” to evaluate the improvement of emotion. The regression models were established with the park characteristics and behavior characteristics as dependent variables, and the causal relationships between these factors were discussed.

## 3. Results

### 3.1. Descriptive Statistics

Test data from 92 older adults can be used for subsequent analysis. The participants included 49 women (53.3%) and 43 men (46.7%), covering 62–81 older adults (Mean = 70.29, SD = 4.766). In China, older adults are generally divided into three age groups: 60–69 young older adults, 70–79 middle-aged older adults, and >79 the very older adults, because this is more in line with their life and psychological state [53]. Therefore, according to this standard, this study divided the age stage, and the sample of 60–69 and 70–79 older adults accounted for 51.1% and 44.6%. Among them, the proportion of women in the 60–69, 70–79, and >79 age groups were 55.3%, 51.2%, and 50%, respectively. Generally speaking, the sex ratio of all the subjects and the age ratio bounded by 70 years old are relatively balanced. In order to further discuss the influencing factors, the investigation of physical health status, living conditions, and living cities were added. Physical health status is measured by “whether suffering from chronic diseases”. Living conditions contain “1 = living with spouse”, “2 = living with spouse and children”, “3 = living with children” and “4 = living alone”, of which older adults living alone account for 10.9%. Living cities can be divided into “1 = located”, meaning long-term living, and “0 = relocated”, meaning floating with their children. In other words, local older adults and the floating older adults account for 68.5% and 31.5%, respectively (Table 3).

Only one of these six sample parks (16.7%) has no water, and three parks (50%) have structures with a good environment. On average, the total area, total path length, square area, fitness equipment space area, and evergreen vegetation area of these sample parks are 24.927 ha, 2.795 km, 0.82 ha, 0.463 ha and 1.102 ha on average (Table 3). Among them, there are 2 parks with a total area > 20 ha and 1 park with total area <10 ha. There are 4 with total path length <2 km, and two parks with square area <0.5 ha and >1 ha, respectively. Meanwhile, there are two 3 and 1 parks with instrument areas of <0.4, 0.4–0.6 and >0.6 ha, respectively, 3 and 1 parks with 1 ha and >2 ha evergreen vegetation area.

Combined with the interview, these participants reported 25 kinds of behavior contents, of which 57 (61.9%) mentioned fitness behaviors mainly including walking, skating, and activities relying on sports equipment; 24 people (26.1%) mentioned entertainment behaviors mainly involving dancing; 11 people (12.0%) mentioned leisure behaviors such as playing cards, playing chess, and accompanying their grandchildren. The average stay time and MVPA time of participants were 42.8043 min (Min = 26, Max = 70, SD = 9.812) and 19.87 min (Min = 0, Max = 46, SD = 13.667) (Table 3).

### 3.2. Statistics of Experimental Results

#### 3.2.1. Cognition Recovery Results

**Figure 4:**
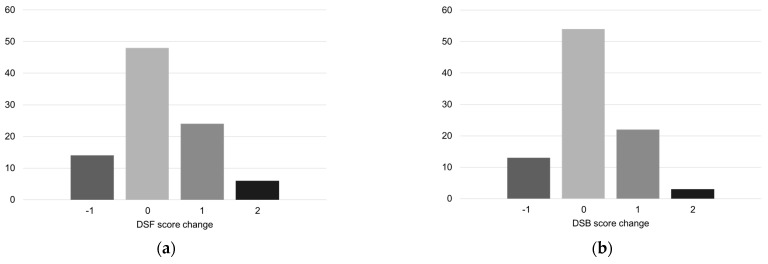
(**a**) DSF test results; (**b**) DSB test results.

Based on the paired sample *t*-test in SPSS, the results indicated that the attention scores of older adults in these parks in winter were significantly improved (Table 4). In the DSF test, the results of 30 older adults have improved, and the average score increased from 5.15 to 5.39. The DSB test showed that the scores of 25 older adults have improved, and the average score has increased from 3.45 to 3.61 (Figure 4).

#### 3.2.2. Emotion Improvement Results

**Figure 5:**
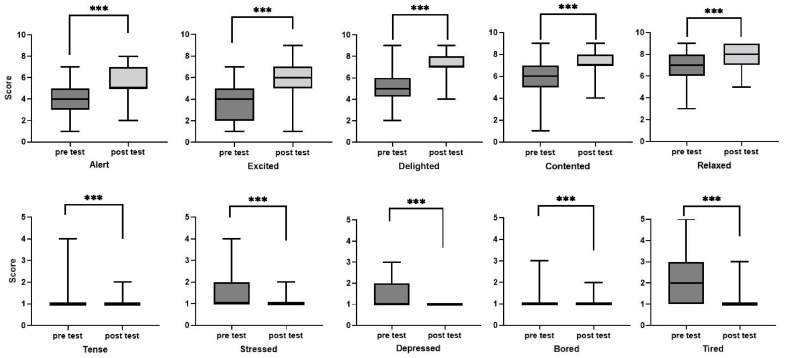
Box chart of winter emotion test results. (*** *p* < 0.01).

The reliability of the questionnaire was analyzed before the emotional test analysis, and Cronbach α reached 0.759, indicating that the reliability is high and can be analyzed in the next step. After comparison, it was found that the post test scores of positive and negative emotions had significant changes compared with the pre-test scores. In the post-test, almost all the negative emotions of older adults were improved to the level of “not at all”. Among positive emotions, “excited” and “delighted” had the highest degree of improvement, while “stressed”, “depressed” and “tired” negative emotions had the most obvious improvement (Figure 5).

### 3.3. Individual Level Analysis: Differences in Mental Health Restorative Effects of Different Gender, Age, Living, Physiological Health and Customary

Independent sample *t*-test and ANOVA analysis were used to test differences of mental health restorative effects of different individuals. The results illustrated that the improvement of “alert” (T = −2.273, sig. = 0.025) and the remission of “tense” were more obvious in women than in men (T = 1.761, sig. = 0.083). In different age stages, 70–79 was stronger in “alert” (F = 6.687, *p* = 0.002), >79 is stronger in “delighted” (F = 4.803, *p* = 0.010) promotion, and 60–69 was better in “stressed” relief (F = 3.112, *p* = 0.049). In the comparison of physical health status, sub-healthy older adults had more significant effects than healthy ones on DSF (T = 2.490, sig. = 0.015), DSB (T = 3.534, sig. = 0.001) and the alleviation of negative emotions. Compared with located and relocated older adults, “tense”, “bored”, “depressed”, and “tired” mitigation effects on older adults who floated with children were stronger. Comparing different living conditions, it was found that older adults who “living with spouse and children” and “living with children” had better mitigation effects in “tense” and “stressed”, and those who “living alone” had stronger recovery effects in “tired”. In addition, by comparing their frequency of visiting the parks, the group that visit the parks with a high frequency had a lower increase in “excited” (F = 3.038, sig. = 0.033), “delighted” (F = 3.969, sig. = 0.011) and “contented” (F = 4.133, sig. = 0.009) than the group that visit the parks with a low frequency.

### 3.4. Park Level Analysis: Differences in Mental Health Restorative Effects of Different Park Types and Characteristics

In the comparison of different types of parks, it was found that in DSB and positive emotion improvement, the effects were “comprehensive park > community park > square”, and in terms of the relief of “stressed”, “depressed”, and “tired”, the restorative effects of comprehensive parks were stronger (Table 5).

Furthermore, it further tested the differences in restoration effects of different park characteristics. First of all, regression models were established for overall cognitive recovery, emotional improvement, and park characteristics, respectively. The results showed that the park characteristics that significantly affected cognitive recovery were the area of evergreen vegetation and the existence of structures; the park characteristic that significantly affected mood improvement was the length of main paths (Table 6). Furthermore, the differences of DSF, DSB, and various emotions in different parks with different characteristics were also investigated. The results reflected that there were significant differences in DSB, and all emotion except “tense” improvement in total park area and total main pathway length. Total square area had significant differences in the improvement of “alert”, “excluded”, “delighted”, “content”, “relaxed”, “stressed”, “bored”, and “depressed”. Total evergreen vegetation area was significantly increased, except for DSF. Besides these factors, parks with water had more significant effects on the improvement of “alert”, “relaxed” and “tense”, “stressed” and “depressed”. In parks with good structure space, DSB, “alert”, “contented”, “relaxed” and negative emotions improved significantly (Table 7).

### 3.5. Behavior Level Analysis: Differences in Restorative Effects of Mental Health with Different Behaviors

Similarly, from the regression model established for cognitive recovery, emotional improvement, and behavioral characteristics (Table 8), it can be seen that total stay time can positively affect cognitive recovery, and total MAVP time can positively affect emotional recovery. Furthermore, using Pearson analysis, total stay time was significantly related to the “stressed” and “tired” relief. Total MVPA time correlated with the improvement of “relaxed”, “tense”, “bored”, “depressed”, and “tired” significantly (Table 9); there was no significant difference in the recovery effect of different behavior types based on the purpose of exercise. Additionally, total stay time was significantly different in parks with different main road lengths, instrument areas, evergreen vegetation areas, and structures. The total MVPA time reflected significance except for the presence of water (Table 10).

## 4. Discussion

### 4.1. Effects on Cognitive Recovery and Emotion Improvement of Older Adults in Winter Parks

This research identified that, in the winter, parks had restorative effects on both attention and emotion. In the experiment of paying attention, DSF improvement was more obvious, which might be related to the greater difficulty of improving DSB. As for the emotional experiment, the positive emotions “excited” and “delighted” with higher arousal were significantly improved, and the negative emotions “tired” with lower arousal were more relieved. These findings were partially consistent with previous studies [21,54,55,56,57]. The existing research showed that the perceived restorative environmental quality is related to the increase in positive emotions and happiness and the decrease in negative emotions [58]. As the essential natural space in cities, the psychological benefits of parks are beyond doubt. ART and SRT may provide some additional explanations, because it points out that a more natural environment often provides the sense of being away, fascinated, coherent and compatible recovery experience [13,58]. In particular, these are similar to the results of a few winter experiments [59].

### 4.2. Individual, Park, Behavior Characteristics and Mental Restorative Effects

#### 4.2.1. Individual Conditions of Older Adults and Mental Restorative Effects

The results indicated that individual conditions except for physical health had no significance on cognition recovery, while there were significant differences in some emotional improvements. Female older adults were more obvious at “alert” promotion and “tension” relief, which might be related to the fact that women play a composite role in families and have more housework and parenting responsibilities, so their prevalence of psychological problems such as stress is higher [60,61]. In different age stages, the improvement of “alert”, “delighted” and “stressed” were stronger for 70–79, >79, and 60–69 older adults, respectively, which correlated to psychological status, psychological elasticity, gender ratio, and more activities in different age groups [62,63]. On the one hand, in China, the retirement age for the general female elderly is 55, and that of the male elderly is 60. Compared with 70–79, the aged 60–69 have just left work life, and their cognitive and responsiveness abilities are strong, so they are relatively low in the promotion of “alert”. Meanwhile, they have just experienced retirement, and most of them have the responsibility to help their children take care of their grandchildren, so their pressure is more obvious. And as for the aged >79 was stronger in “delighted” promotion, it may be due to the older the elderly, the stronger their psychological resilience. They have stronger self-digestibility of negative emotions, so there is no difference in the restorative effect of negative emotions. On the other hand, it may be due to the larger proportion of women in 60–69 and the fact that women are more willing than men to visit parks [64]. In terms of physical health status, the recovery of attention and the improvement of negative emotions in sub-healthy older adults were better. Existing studies have demonstrated that physical health is significantly related to self-rated health [39,40]. In a living city, “tense”, “bored”, “depressed”, and “tired” effects on relocated older adults were stronger. Relocated means leaving original living environment and floating with their children. Thus, such older adults face more challenges such as integration and adaptation, which have a negative impact on their physical, mental, and social health. Comparing different living conditions, older adults who “live with their spouse and children” or “live with their children” have better mitigation effects in “tense” and “stressed”, and ones who “live alone” have stronger recovery effects in “tired”. Existing studies have revealed that the mental health levels of those who live alone are poor [63]. In China, it is very common for older adults to look after their grandchildren, which virtually increases the pressure on them. This may explain these analysis results from another aspect. Moreover, compared with park visit frequency, parks had better emotional improvement for older adults who do not often visit parks. This may be because older adults who often visit parks are more familiar with the park environment, and the attraction and arousal of the environment are not obvious.

#### 4.2.2. Park Characteristics and Mental Restorative Effects

For one thing, there was no significant difference among different park types in DSF in winter, while comprehensive parks performed better in DSB and emotional improvement and squares had the lowest restorative effect. It may be that squares with large proportion of hard pavement have a cold vision and feeling. These results were consistent with previous research results on forest therapy to some degree [54,55,56].

For another, it was found that park characteristics that significantly affected cognitive recovery were evergreen vegetation area and structures, while the length of main paths significantly affected emotional improvement. The reasons for these results may be: Firstly, the area of evergreen vegetation can bring a living visual sense, which is similar to the use of green in healing spaces; secondly, spaces with structure often carry playing cards and chess, which can help them focus their attention, so the cognitive recovery effect is better; Thirdly, older adults mainly walk and jog in parks in the winter. Roe, et al. [18] also proved the relationship between walking and emotion, which also verified the results of this study to some extent.

In the performance of cognition and emotion, DSB and most of the mood improved with a larger park area, main pathway length, equipment area, square area, and evergreen vegetation area. Besides that, parks with water performed better on “alert”, “relaxed” and “tense”, “stressed”, and “depressed” improvement. This was similar to the existing research conclusion that water can promote positive emotions in viewers. In parks with structures, it was better to improve DSB score, “alert”, “contented”, “relaxed” and negative emotions. One possible explanation is that nature is very important to older adults, and it can bring direct recovery effect for elderly adults. [65]. Even in winter, as long as older adults are exposed to nature outdoors, no matter what behavior they carry out, it will have restorative effects [59]. Particularly, evergreen space to some extent represents the restoration potential of natural space in non-winter. Jiang, et al. [50] showed that the enhancement of pressure recovery is related to areas with higher tree coverage, which may be related to a more natural environment. Another possibility is that different park design features will affect the length of stay time and the occurrence of older adults’ different behaviors and then affect the restorative effect.

#### 4.2.3. Use Behaviors, Park Characteristics and Mental Restorative Effects

This study investigated the relationships between use behaviors, restorative effects, and park characteristics. It was found that park stay time and total MVPA time can positively affect cognitive recovery and emotional improvement, respectively. The longer stays, the better the “stressed” and “tired” mitigation effects. Total MVPA time was positively correlated with the improvement of “relaxed”, “tense”, “bored”, “depressed”, and “tired”. Among them, parks with a larger main pathway length, equipment space area, evergreen vegetation area, and structures lasted a longer time. For parks with large, continuous variables in their design features and with water, the MVPA time was longer. However, there was no significant difference between different behavior types and recovery effects.

In this research, based on the results of GPS locators and interview results, older adults stayed in the parks for 48 min and engaged in MVPA for 19.870 min on average, both of which were less than 57 min and 32.73 min in Zhai, et al.’s research [31]. These results mainly depend on the location and month of the experiment. Compared with September in Guangzhou, the winter climate in Changchun is cold, so the stay time and MVPA time of older adults are relatively low. In addition, this is related to older adults visiting the park mainly for physical exercise. Researchers showed that older people in Asia were more likely to exercise in parks, while older people in Western countries were more likely to engage in recreational and relaxation behaviors [16,66,67,68].

The difference and correlation analysis results of park characteristics and behavior characteristics confirmed the two possible reasons mentioned above. To put it another way, park characteristics may have both direct recovery effects and indirect effects through influencing behavior in elderly adults. First, the greater the exploration space for older adults, and the longer the stay time and MVPA time. Second, this study found that the total length of the park path (width >3.5 m) was positively correlated with stay time and MVPA among older adults. This result is consistent with existing findings from adults that paths can encourage physical activity [8,44,45]. For older adults, walking is the most popular park-based physical activity [66,67,69]. Older adults prefer to carry out more intensive activities on wider paths, such as fast walking and jogging, which is more obvious in the winter. A recent study in Shanghai also found seniors in neighborhood parks with longer trails walk more steps during park visits [36]. Third, square area was positively correlated with stay time and MVPA time in this study. Square space is another space preferred by older adults. What is different from Zhai, et al. ‘s [31] research is that in winter squares, people engage in more dancing and other behaviors, and exercise intensity is higher. Moreover, dancing will attract more elderly people to watch and create greater attraction. Fourth, different equipment space area did not affect the length of stay time, but related to MVPA time. This is partially consistent with the conclusions of previous studies [44,70]. Fifth, a larger evergreen area may increase the stay time and MVPA time to some extent. On the one hand, this is related to the visual sense of vitality brought by evergreen plants in winter, which is similar to the biological property. On the other hand, evergreen plants play a certain role in purifying the air and isolating noise, which can help create a more comfortable atmosphere for activities.

Additionally, the behaviors relying on structures are mostly leisure behaviors, such as playing cards, which last for a long time. Activities relying on water are usually moderate-intensity sports, such as skating, so in winter, the presence of water will affect the intensity of sports. Simultaneously, the longer stay time in parks, the higher activity level. This has also been confirmed in studies of community parks and older adults [31,71]. It is worth mentioning that these three behavior types cover all the contents with different exercise intensities. Consequently, there was no significant difference in the restorative effects of behavior types divided solely by the purpose of exercise.

### 4.3. Limitations

This study has some limitations that should be considered. For one thing, although the sample parks covered comprehensive parks, community parks, and squares, the data volume was limited due to the difficulty in obtaining data, and the results also had some limitations. For another, although six representative parks were investigated, all of them are located in the central urban area of Changchun, China. In urban areas, suburbs, and rural areas with low population density and other cultures, park characteristics and park use patterns may be different. Furthermore, for the older adults’ group, the difficulty of the experiment had increased a lot, especially in winter in severe cold areas. In order to ensure the enthusiasm of participants and the effectiveness of the data, we simplified the experimental process as much as possible. In the future, the real-time relationship between special people and space can be further explored in combination with heart rate monitors or multi-sensor devices.

## 5. Conclusions

The purpose of this study was to discuss whether cold city parks have short-term mental restorative effects on older adults in winter and to explore the potential factors leading to differences in these effects. The results indicated that, on the one hand, even in winter, parks in cold regions still have cognitive recovery and emotional relief effects on older adults. On the other hand, individual conditions, park characteristics, and use behavior will affect these restoration effects to varying degrees.

First, individual conditions were related to these effects. For example, there were significant differences in female’s “alert” improvement, and certain emotional performance at different ages; the more obvious the restorative effects were for the sub-health participants compared with the healthy ones; these effects for older adults living alone and floating with their children were more obvious; and some emotional improvements for those who do not often visit parks were stronger. Second, total park area, main pathway length, square area, equipment space area, evergreen vegetation area, and the presence of water and structures were positive predictors of restorative effects on older adults’ mental health. Third, the area of evergreen vegetation and the structures were the main factors affecting cognitive recovery, and the length of main pathway was the main factor affecting emotional improvement. The behavioral characteristic factors that mainly affect these two effects are stay time and MVPA time, respectively. Besides these, the length of stay in these parks was positively correlated with “stressed” and “tired” relief. MVPA time was correlated with the improvement of “relaxed”, “tense”, “bored”, “depressed” and “tired” positively. Stay time was related to the length of the main pathway, the area of instruments, evergreen and the existence of structures; MVPA time was related to the park areas, main pathway lengths, equipment areas, square areas, and the presence of water.

These findings can be used to guide the design and management of parks, so as to maximize restorative effects on older adults’ mental health. For instance, planners and designers can appropriately increase the natural vegetation area and reduce the impervious area, create longer footpaths, and provide appropriate squares, equipment spaces, well-landscaped water, and structures on the basis of the winter research conclusion. In future design and practice, we should also consider more carefully the impact of spatial characteristics under different perception dimensions on mental health recovery for older adults.

## Figures and Tables

**Table 1:** Basic information of sample parks.

Park Name	District	Park Type	Year of Construction	Area (ha)
CC Park	Lvyuan District	Comprehensive Park	1999	66
SL Park	Kuancheng District	Comprehensive Park	1915	24.5
LD Park	Erdao District	Comprehensive Park	1936	16.5
DS Park	Qikai District	Community Park	1997	8.5
JJ Park	Qikai District	Square	2003	18.8
KC Park	Kuancheng District	Square	2007	15.26

Source: Open data query from Bureau of Forestry and Landscaping of Changchun.

**Table 2:** Park design characteristics and behavior characteristics.

		Variable Type	Data Source
Park characteristics	P1: Total area (ha)	Categorical (<10 ha, 10–20 ha and >20 ha)/Continuous	Auto CAD map/site visit
	P2: Total pathway (width >3.5 m) length (km)	Categorical (<1 km, 1–2 km and >2 km)/Continuous	
	P3: Total square (>1000 m^2^) area (ha)	Categorical (<0.5 ha, 0.5–1 ha and >1 ha)/Continuous	
	P4: Total fitness equipment space area (ha)	Categorical (<0.4 ha, 0.4–0.6 ha and >0.6 ha)/Continuous	
	P5: Evergreen vegetation area (ha)	Categorical (<1 ha, 1–2 ha and >2 ha)/Continuous	
	P6: Presence of water	Categorical (0 = without, 1 = with)	
	P7: Presence of structure	Categorical (0 = without, 1 = with)	
Behavior characteristics	B1: Park stay time (min)	Continuous	Pedometer/ behavior observation
	B2: Total MVPA time (min)	Continuous	
	B3: Behavior type	Categorical (1 = fitness, 2 = entertainment, 3 = leisure)	Questionnaire

**Table 3:** Descriptive statistics for senior participants, parks and behavioral characteristics.

		Frequency	Percent			Frequency	Percent
S1: Gender	1. Male	43	46.7	S3: Suffering from chronic diseases	1. Yes	50	54.3
	2. Female	49	53.3		0. No	42	45.7
S2: Age	1. 60–69	47	51.1	S4: Living conditions	1. Living with spouse	61	66.3
	2. 70–79	41	44.6		2. Living with spouse and children	11	12.0
	3. >79	4	4.3		3. Living with children	10	10.9
S6: Park visit frequency	1. Everyday	18	19.6		4. Living alone	10	10.9
	2. 3–4 times per week	32	34.8	S5: Living city	1. Located	63	68.5
	3. 1–2 times per week	33	35.8		0. Relocated	29	31.5
	4. Very occasionally	9	9.8	P6: Presence of water	0. Without	1	16.7
B3: Behavior type	1. Fitness	57	61.9		1. With	5	83.3
	2. Entertainment	24	26.1	P7: Presence of structure	0. Without	3	50
	3. Leisure	11	12		1. With	3	50
				Min	Max	M	SD
P1: Total area(ha)				8.50	66.00	24.927	20.780
P2: Total pathway (width > 3.5 m) length(km)				1.45	7.22	2.795	2.248
P3: Total square(>1000 m^2^) area(ha)				0.31	1.50	0.820	0.476
P4: Total fitness equipment space area(ha)				0.16	0.75	0.463	0.231
P5: Evergreen vegetation area(ha)				0.18	2.46	1.102	0.923
B1: Park stay time(min)				26.00	70.00	42.804	9.813
B2: Total MVPA time(min)				0.00	46.00	19.870	13.667

**Table 4:** Paired sample *t*-test of older adults’ attention test results in winter park.

		Paired Differences					*t*	df	Sig. (2-Tailed)
		Mean	Std. Deviation	Std. Error Mean	95% Confidence Interval of the Difference				
					**Lower**	**Upper**			
1	DSF score change	0.239	0.790	0.082	0.076	0.403	2.905 **	91	0.005
2	DSB score change	0.163	0.700	0.073	0.018	0.308	2.235 **	91	0.028

** *p* < 0.05.

**Table 5:** Differences in restorative effects of different park types.

	M			F	*p*
	Comprehensive Park	Community Park	Square		
DSF	0.31	0.25	0.05	0.768	0.467
DSB	0.38	0.05	−0.30	8.431	0.000 ***
E1	1.67	0.90	1.55	3.30	0.041 **
E2	2.31	1.75	1.50	3.886	0.024 **
E3	2.29	1.65	1.45	4.876	0.010 **
E4	1.31	0.80	0.65	3.708	0.028 **
E5	1.42	0.60	0.25	10.235	0.000 ***
E6	−0.23	0.00	−0.10	2.192	0.118
E7	−0.58	−0.10	−0.10	6.305	0.003 **
E8	−0.40	−0.20	−0.10	2.374	0.102
E9	−0.62	−0.15	−0.25	4.612	0.012 **
E10	−0.96	−0.65	0.00	6.174	0.003 **

** *p* < 0.05 (2-tailed). *** *p* < 0.01 (2-tailed).

**Table 6:** Cognitive recovery, emotional improvement and park characteristics (stepwise model).

	Variables	Coef. (B)	SE	St. Coef. (β)	*t*	Sig.	Overall Model
Cognitive recovery	(constant)	−0.339	0.170		−1.996	0.049	R^2^ = 0.095 Sig. = 0.026
	P5	0.218	0.078	0.279	2.779	0.007	
	P6	0.262	0.116	0.227	2.266	0.026	
Emotional improvement	(constant)	0.319	0.082		3.872	0.000	R^2^ = 0.104 Sig. = 0.001
	P2	0.383	0.045	0.669	8.549	0.000	

**Table 7:** Differences in restorative effects of different park characteristics.

		M			F	*p*		M			F	*p*
		<10	10–20	>20				<10	10–20	>20		
P1	DSF	0.25	0.19	0.28	0.101	0.904	DSB	0.05	−0.06	0.44	5.406	0.006 **
	E1	0.90	1.36	1.92	5.560	0.005 **	E6	0.00	−0.25	−0.14	2.124	0.126
	E2	1.75	1.64	2.53	5.773	0.004 **	E7	−0.10	−0.33	−0.56	3.158	0.047 **
	E3	1.65	1.50	2.61	10.599	0.000 ***	E8	−0.20	−0.14	−0.50	4.013	0.021 **
	E4	0.80	0.72	1.53	6.637	0.002 **	E9	−0.15	−0.36	−0.67	4.298	0.017 **
	E5	0.60	0.64	1.56	7.827	0.001 ***	E10	−0.65	−0.25	−1.14	6.634	0.002 **
P2		<2	2–4	>4	F	*p*		<2	2–4	>4	F	*p*
	DSF	0.21	0.21	0.32	0.142	0.868	DSB	−0.02	0.43	0.45	5.234	0.007 **
	E1	1.20	2.29	1.68	5.732	0.005 **	E6	−0.16	0.00	−0.23	1.156	0.320
	E2	1.68	2.64	2.45	5.833	0.004 **	E7	−0.25	−0.21	−0.77	5.713	0.005 **
	E3	1.55	2.07	2.95	14.198	0.000 ***	E8	−0.16	−0.29	−0.64	5.784	0.004 **
	E4	0.75	0.93	1.91	11.766	0.000 ***	E9	−0.29	−0.36	−0.86	6.424	0.002 **
	E5	0.63	0.79	2.05	15.339	0.000 ***	E10	−0.39	−1.0	−1.23	5.785	0.004 **
P3		<0.4	0.4–0.6	>0.6	F	*p*		<0.4	0.4–0.6	>0.6	F	*p*
	DSF	0.19	0.24	0.32	0.165	0.848	DSB	−0.06	0.21	0.45	3.973	0.022 **
	E1	1.36	1.47	1.68	0.499	0.609	E6	−0.25	0.00	−0.23	3.364	0.039 **
	E2	1.64	2.12	2.45	3.338	0.040 **	E7	−0.33	−0.15	−0.77	6.536	0.002 **
	E3	1.50	1.82	2.95	13.502	0.000 ***	E8	−0.14	−0.24	−0.64	5.761	0.004 **
	E4	0.72	0.85	1.91	11.725	0.000 ***	E9	−0.36	−0.24	−0.86	6.726	0.002 **
	E5	0.64	0.68	2.05	15.172	0.000 ***	E10	−0.25	−0.79	−1.23	6.333	0.003 **
P4		<0.5	0.5–1	>1	F	*p*		<0.5	0.5–1	>1	F	*p*
	DSF	0.30	0.17	0.25	0.215	0.807	DSB	0.33	−0.03	0.19	2.142	0.123
	E1	1.67	1.33	1.44	0.621	0.311 **	E6	−0.23	−0.03	−0.19	1.710	0.187
	E2	2.20	1.57	2.25	3.016	0.054 *	E7	−0.43	−0.10	−0.56	4.097	0.020 **
	E3	1.80	1.57	2.50	5.744	0.005 **	E8	−0.23	−0.13	−0.50	3.461	0.036 **
	E4	0.87	0.73	1.53	5.569	0.005 **	E9	−0.43	−0.20	−0.66	3.642	0.030 **
	E5	0.97	0.50	1.47	5.760	0.004 **	E10	−0.77	−0.37	−0.91	2.036	0.137
P5		<1	1–2	>2	F	*p*		<1	1–2	>2	F	*p*
	DSF	0.15	0.34	0.21	0.579	0.562	DSB	−0.13	0.37	0.43	6.804	0.002 **
	E1	1.23	1.45	2.29	4.530	0.013 **	E6	−0.05	−0.32	0.00	0.4.859	0.010 **
	E2	1.63	2.18	2.64	4.471	0.014 **	E7	−0.10	−0.71	−0.21	10.095	0.000 ***
	E3	1.55	2.37	2.07	5.090	0.008 **	E8	−0.15	−0.45	−0.29	2.614	0.079 *
	E4	0.73	1.45	0.93	5.029	0.009 **	E9	−0.20	−0.71	−0.36	6.157	0.003 **
	E5	0.43	1.66	0.79	13.948	0.000 ***	E10	−0.33	−0.95	−0.10	4.062	0.021 **
P6		1	0		T	Sig.		1	0		T	Sig.
	DSF	0.24	0.25		−0.069	0.945	DSB	0.19	0.05		0.815	0.417
	E1	1.64	0.90		2.549	0.013 **	E6	−0.19	0.00		−3.345	0.001 ***
	E2	2.08	1.75		1.068	0.288	E7	−0.44	−0.10		−3.128	0.003 **
	E3	2.06	1.65		1.354	0.179	E8	−0.32	−0.20		−0.807	0.422
	E4	1.12	0.80		1.213	0.228	E9	−0.51	−0.15		−2.639	0.011 **
	E5	1.10	0.60		1.682	0.096 *	E10	−0.69	−0.65		−0.159	0.874
P7		1	0		T	Sig.		1	0		T	Sig.
	DSF	0.31	0.12		1.132	0.261	DSB	0.26	0.00		1.730	0.087 *
	E1	1.26	1.85		−2.389	0.019 **	E6	−0.21	−0.06		−1.855	0.067 *
	E2	2.03	1.97		0.238	0.812	E7	−0.50	−0.15		−2.958	0.004 **
	E3	2.12	1.71		1.628	0.107	E8	−0.36	−0.18		−1.688	0.095 *
	E4	1.22	0.76		2.287	0.025 **	E9	−0.52	−0.29		−1.668	0.099 *
	E5	1.29	0.47		3.784	0.000 ***	E10	−0.84	−0.41		−1.849	0.068 *

* *p* < 0.10 (2-tailed). ** *p* < 0.05 (2-tailed). *** *p* < 0.01 (2-tailed).

**Table 8:** Cognitive recovery, emotional improvement and behavior characteristics (stepwise model).

	Variables	Coef. (B)	SE	St. Coef. (β)	t	Sig.	Overall Model
Cognitive recovery	(Constant)	−0.391	0.362		−1.080	0.283	R^2^ = 0.061 Sig. = 0.061
	P1	0.013 **	0.006	0.227 **	2.147	0.035	
Emotional improvement	(Constant)	0.323	0.308		1.050	0.297	R^2^ = 0.087 Sig. = 0.017
	P2	0.009 **	0.004	0.254 **	2.455	0.016	

** *p* < 0.05.

**Table 9:** Correlation of stay time, MVPA time and restorative effects.

	B1	B2		B1	B2
DSF	0.148	−0.151	DSB	0.147	−0.014
E1	−0.134	0.039	E6	−0.141	−0.210 **
E2	0.112	0.053	E7	−0.237 **	−0.080
E3	−0.012	0.108	E8	−0.091	−0.213 **
E4	0.150	0.061	E9	−0.072	−0.178 *
E5	0.138	0.328 ***	E10	−0.206 **	−0.185 *

* *p* < 0.10 (2-tailed). ** *p* < 0.05 (2-tailed). *** *p* < 0.01 (2-tailed).

**Table 10:** Restorative effects differences of different stay time, MVPA time and park characteristics.

	P1	P2	P3	P4	P5	P6	P7
B1	0.920	3.840 **	3.494 **	0.797	11.393 ***	0.028	4.883 ***
B2	3.294 **	3.505 **	3.347 **	4.219 **	2.952 *	1.961 *	0.560

* *p* < 0.10 (2-tailed). ** *p* < 0.05 (2-tailed). *** *p* < 0.01 (2-tailed).

## Data Availability

Data sharing not applicable to this article as no datasets were generated or analysed during the current study.
